# Pneumonia

**Published:** 1854-06

**Authors:** 


*Pneumonia.* Its supposed connection pathological and etiological, with autumnal fevers: including an inquiry into the existence and morbid agency of Malaria, by R. LaRoche, M.D., Member of the American Philosophical Society, &c., &c. Philadelphia, Blanchard & Lea, 1854.

This book is a valuable addition to the home literatare of our profession. We have not time or space in the present Number of the Journal, to do more than acknowledge its receipt, and give a synopsis of its table of contents.

In chapter first, the author discusses the belief so long entertained, that a relation does exist between the two classes of disease.

Chapters second, third and fourth, are devoted to a consideration of the question, as to the existence and morbid agency of Malaria.

In chapter fifth, Pneumonia and Autumal fevers are compared, in reference to their causes, mode of progression, symptoms, anatomical characters, and the circumstances by which they are influenced.

In chapter sixth, Pneumonia and Autumnal fevers are compared, in reference to the powers of acclimatization, ages, sexes, and races affected; and the prevalance of the two diseases at the same time, or in rapid succession, shown to be no proof of identity.

In chapter seventh, it is shown that Pneumonia and Autumnal fevers, although independent of each other as regards nature and cause, combine together and form like other complaints hybrid diseases, which must not be considered as peculiar forms of either.

This chapter completes the work At some future time we promise ourselves and our readers the pleasure of a review In the meantime, we must thank Dr. LaRoche in the name of the profession, for the very valuable service which he has rendered to our science in the work before us. J.

For Sale by D. B Cooke & Co., 135, Lake Street, Chicago.

